# Recent Outbreaks of Rift Valley Fever in East Africa and the Middle East

**DOI:** 10.3389/fpubh.2014.00169

**Published:** 2014-10-06

**Authors:** Yousif E. Himeidan, Eliningaya J. Kweka, Mostafa M. Mahgoub, El Amin El Rayah, Johnson O. Ouma

**Affiliations:** ^1^Entomology Unit, Faculty of Agriculture and Natural Resources, University of Kassala, New Halfa, Sudan; ^2^Africa Technical Research Centre, Vector Health International, Arusha, Tanzania; ^3^Division of Livestock and Human Diseases Vector Control, Tropical Pesticides Research Institute, Arusha, Tanzania; ^4^Department of Medical Parasitology and Entomology, Catholic University of Health and Allied Sciences, Mwanza, Tanzania; ^5^Blue Nile National Institute for Communicable Diseases, University of Gezira, Madani, Sudan; ^6^Department of Zoology, University of Khartoum, Khartoum, Sudan; ^7^Biotechnology Research Institute, Kenya Agricultural and Livestock Research Organization, Kikuyu, Kenya

**Keywords:** RVFV outbreaks, *Aedes* mosquitoes, rainfall, East Africa

## Abstract

Rift Valley fever (RVF) is an important neglected, emerging, mosquito-borne disease with severe negative impact on human and animal health. Mosquitoes in the *Aedes* genus have been considered as the reservoir, as well as vectors, since their transovarially infected eggs withstand desiccation and larvae hatch when in contact with water. However, different mosquito species serve as epizootic/epidemic vectors of RVF, creating a complex epidemiologic pattern in East Africa. The recent RVF outbreaks in Somalia (2006–2007), Kenya (2006–2007), Tanzania (2007), and Sudan (2007–2008) showed extension to districts, which were not involved before. These outbreaks also demonstrated the changing epidemiology of the disease from being originally associated with livestock, to a seemingly highly virulent form infecting humans and causing considerably high-fatality rates. The amount of rainfall is considered to be the main factor initiating RVF outbreaks. The interaction between rainfall and local environment, i.e., type of soil, livestock, and human determine the space-time clustering of RVF outbreaks. Contact with animals or their products was the most dominant risk factor to transfer the infection to humans. Uncontrolled movement of livestock during an outbreak is responsible for introducing RVF to new areas. For example, the virus that caused the Saudi Arabia outbreak in 2000 was found to be the same strain that caused the 1997–98 outbreaks in East Africa. A strategy that involves active surveillance with effective case management and diagnosis for humans and identifying target areas for animal vaccination, restriction on animal movements outside the affected areas, identifying breeding sites, and targeted intensive mosquito control programs has been shown to succeed in limiting the effect of RVF outbreak and curb the spread of the disease from the onset.

## Introduction

Rift Valley fever (RVF) is an important neglected, emerging, mosquito-borne disease with severe negative economic impact as it affects human and animal health. The disease is caused by RVF virus (RVFV) an acute febrile arbovirus in the *Phlebovirus* genus and Bunyaviridae family. The disease was first characterized by Daubney et al. (1) while working at the Veterinary Research Laboratory at Kabete in Kenya. An earlier report by Stordy (2) had described a similar disease syndrome, which may well have been RVF, it was described as an acute and highly fatal disease in the Rift Valley in exotic wool sheep, which had been imported into East Africa from Europe (1, 3). These European stock species were more severely affected than native African stock. The disease remained a veterinary concern in East Africa until a major outbreak occurred in Egypt in 1977. A second outbreak outside East Africa occurred in 2000 when RVF moved into Saudi Arabia and Yemen in the Arabian Peninsula (4). This was the first time the disease was being detected outside of Africa – where it had been confined so far – becoming a threat to the Middle East.

From the most recent outbreaks that occurred in Kenya, Somalia, Tanzania in 2007 (5, 6), and Sudan in 2008 and 2010 (7, 8), RVF appears to have great potential for spreading into new areas and with huge impact on human and animal health. This calls for an integrated approach between different governmental sectors and organizations within and between countries and regions to address both human and animal health. Limited information is available on the evolution of RVF between East Africa and Middle East. In order to highlight the urgent need of establishing a health system for controlling RVF in the region, this review article aims to gather experiences and highlight basic information on the ecological aspects, epidemiological, and risk factors associated with the distribution of recent outbreaks in East Africa and Middle East.

## Transmission and Impact

The virus is known to infect a range of animal hosts including sheep, cattle, goats, camels, buffaloes, and others. The incubation period in animals is between 1 and 6 days in general, 1 and 3 days in sheep, and only about 12 and 36 h in lambs (9). Sheep and to a lesser extent cattle were the principle disease hosts in both East and southern Africa (3). Sheep seemed to be the most susceptible animal as it was noted that RVF caused high rates of abortions during pregnancy and high mortalities among newborns (10, 11). Lambs can die before they acquire passive immunity and mortality and abortion rates among old sheep range from 5 to 100% (9). Infections can therefore cause severe disease and result in significant economic losses. For example, the 2007 outbreak was the most widespread affecting livestock in 11 regions in Tanzania and Kenya. A total of 16,973 cattle, 20,193 goats, and 12,124 sheep died of the disease, with spontaneous abortions reported in 15,726 cattle, 19,199 goats, and 11,085 sheep (12, 13). Considering the wide-ranging impacts of the disease on the livestock sector and other segments of the economy, the 2007 RVF outbreak in Kenya alone induced losses of over Ksh 2.1 billion (US$32 million) on the Kenyan economy (14). The overall economic loss in East Africa is estimated to exceed $60 million because of disruption in trade from these recent epizootics between 2006 and 2007 (15). In Saudi Arabia, during the outbreak of 2000, it was estimated that around 40,000 animals including sheep, goats, camels, and cattle died whereas 8,000–10,000 of them aborted (16). The outbreaks of 2007 in Sudan led to bans in livestock trade between Saudi Arabia and Sudan, resulting in vast economic impact on the animal market in the two countries (17).

**Figure 1:**
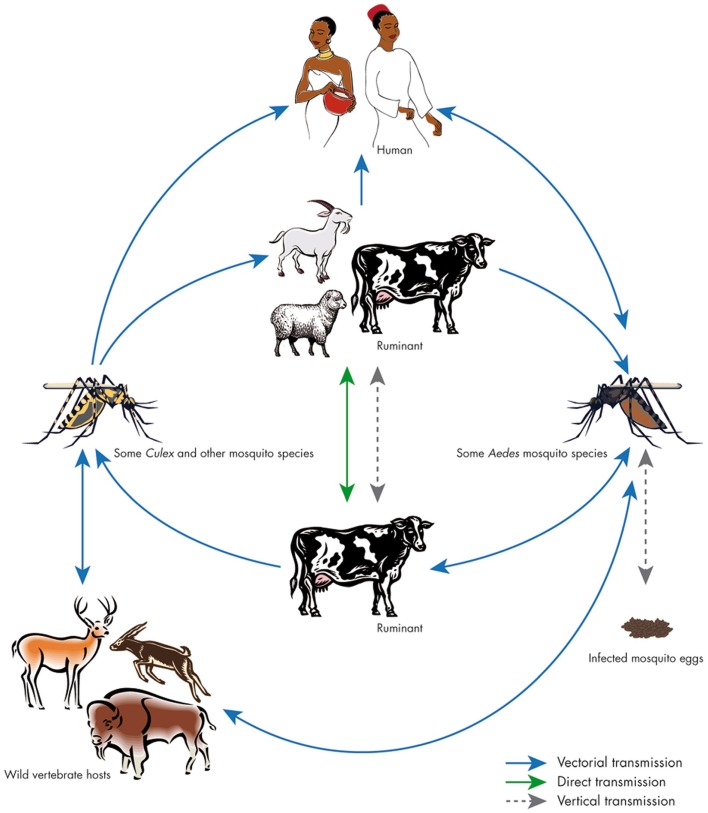
**Cycle of Rift Valley fever**. The virus can be maintained in an enzootic cycle involving *Aedes* mosquitoes, which are able to transmit the virus vertically to their offspring. Epizootic outbreaks are often linked with unusual rains or warm seasons, favoring the hatching of infected *Aedes* eggs that are then able to initiate the virus circulation. Subsequently, large numbers of secondary vectors belonging to the *Culex* genus could be infected and induce the emergence of epidemic/epizootic outbreaks. Transmission to humans occurs through direct contact with high-virus loads when aborted fetuses are manipulated. Source: Balenghien et al. (34), with permission from Thomas Balenghien, CIRAD, UMR Contrôle des maladies, F-34398 Montpellier, France.

Infection by RVF usually spreads among livestock first through mosquitoes bites. In addition, the infection can also be transmitted vertically between animals (18) (Figure 1). From domestic animals, the virus is transmitted to humans mainly through direct contact with blood, excreta, meat, or secretions of infected animals, consumption of raw milk (19–21), and in few cases, transmission through mosquito bites that belong to the genera *Anopheles, Aedes*, and *Culex* seems to occur (22, 23) (Figure 1). Symptoms of RVF in humans vary from a flu-like syndrome to encephalitic, ocular, or hemorrhagic syndrome. The case fatality rate of the hemorrhagic syndrome form can be as high as 50% (24). The most severe outbreaks of 1997–1998 and 2006–2007 in Tanzania, Kenya, and Somalia caused 478 human deaths in 1998 and 309 in 2007 (25–27). The outbreak of 2000 resulted in 883 human cases with 124 deaths (case fatality rate, 14%) in Saudi Arabia (28) and 1,328 human cases, with 166 deaths in neighboring north western Yemen (29–31). In Sudan, the outbreak of 2007 resulted in 698 cases, including 222 deaths (32, 33).

## Mosquito Vectors

Mosquitoes in the *Aedes* genus have been considered the primary maintenance host and source of RVFV that initiate disease outbreaks (34, 35). RVFV is known to be carried in the eggs of *Aedes* mosquitoes, which can survive for several years in the dried mud (36). On flooding, *Aedes* mosquito species play an important role in initiation of infection and virus circulation. The survival of RVFV during inter epizootics was believed to depend on transovarial transmission of the virus in flood water by *Aedes* mosquitoes (37). Other mosquitoes in the *Culex* and *Anopheles* genus are thought to be important in amplification of virus. activity during outbreaks. The virus has also been detected in phlebotomine sand flies, *Culicoides* midges, and *Amblyomma* tick species although these infections are thought not to play an important role in the life cycle of the virus or in disease outbreak settings (38–42). In the laboratory, the RVFV was also transmitted trophically or mechanically by other hematophagous flies but field relevance of these transmission routes are still unclear (43, 44).

The important RVF vectors in East Africa include *Aedes mcintoshi, Aedes ochraeus, Culex pipiens, Aedes dalzieli*, and *Aedes vexans* (45). Records also indicated that *A. mcintoshi* is the main vector for RVF in Kenya (41, 42, 46). Investigation on RVFV by reverse transcription-polymerase chain reaction (RT-PCR) during the recent outbreak of 2006/2007 in Kenya showed that 77 out of 3,003 pools representing 10 species, from 4 affected districts, tested positive for RVFV, including *A. mcintoshi*/*circumluteolus* (26 pools), *Aedes ochraceus* (23 pools), *Mansonia uniformis* (15 pools); *Cx. poicilipes, Culex bitaeniorhynchus* (3 pools each); *Anopheles squamosus, Mansonia africana* (2 pools each); *Culex quinquefasciatus, Culex univittatus, Aedes pembaensis* (1 pool each). *A. pembaensis, Cx. univittatus*, and *Cx. bitaeniorhynchus* were for the first time observed positive for the virus (42). The observation of infected *A. ochraceus* in Garissa, Kenya, represents a new RVFV-vector association in East Africa. *A. ochraceus* is a known vector of RVFV in West Africa (39), along with *A. vexans arabiensis* and *A. dalzieli*. *A. vexans arabiensis* is also a vector of RVFV in Saudi Arabia (40, 44) and although the species has not been documented in Kenya, it has been found in neighboring Somalia and Sudan (47, 48) (Table 1).

**Table 1:** **Mosquito species incriminated in the transmission of RVFV during the outbreaks recorded in East Africa and the Middle East**.

Year of outbreak	Affected country	Collected mosquitoes	Reference
1997–1998 and 2006	Kenya	*Culex zombaensis, Culex poicilipes, Culex bitaeniorhynchus, Culex quinquefasciatus, Culex univittatus, Anopheles coustani, Anopheles squamosus, Aedes mcintoshi, Aedes ochraceus, Aedes pembaensis Mansonia africana, M. uniformis*	(42, 49)
1997–1998 and 2007	Tanzania	*Aedes mcintoshi*	(6)
1997–1998	Eastern Africa	*Culex theileri*	(50)
1977	Egypt	*Culex pipiens*	(51, 52)
2000	Kingdom Saudi Arabia	*Culex pipiens, Aedes vexans arabiensis, Ae. Vittatus, Ae. (Stegomyia) nilineatus, Aedes vexans arabiensis*, and *Culex triteniorynchus*	(30, 40, 53–55)
2000	Yemen	Not defined	(30, 54, 55)
2007–2008	Sudan	*Cx pipiens, Cx. Poicilipes, An. arabiensis, An. coustani, Ae. aegypti*	(5, 21, 49)
1997–1998 and 2006–2007		Not defined	(6)

It has been suggested that different mosquito species serve as epizootic/epidemic vectors of RVFV in diverse ecologies, creating a complex epidemiological pattern in East Africa (42). *A. aegypti* has been found naturally infected with RVFV and seemed to be the main source of the infection during the outbreak of 2007 in Sudan. During this outbreak, RVFV was successfully detected by RT-PCR in larvae, male and females of *An. arabiensis, An. coustani, Cx. pipiens* complex, *Cx. poicilipes*, and *A. aegypti* (Table 1). The infections were considered as a precursor for viral circulation in these species (incriminated in dissemination or acquired the virus in its mid gut only) (21). The detection of RVFV in male and larval stages indicated transovarial (vertical) transmission of the virus within these mosquito species. It may also show possible venereal RVFV transmission when a male is infected vertically and then infects the female during mating (21).

Laboratory established colonies of *A. aegypti* from Tahiti exhibited the highest infection rates of RVFV when compared with other potential vectors in the Mediterranean region (56). *A. aegypti* has also demonstrated infection and transmission rates of the non-structural proteins (NSs) deletion virus similar to wild-type virus, but dissemination rates were significantly reduced (35). *Cx. pipiens* was incriminated as the main RVF vector in Egypt based on field isolates and also in Maghreb and South Africa based on laboratory experiments (57–59).

## Occurrence of RVF Outbreaks

**Figure 2:**
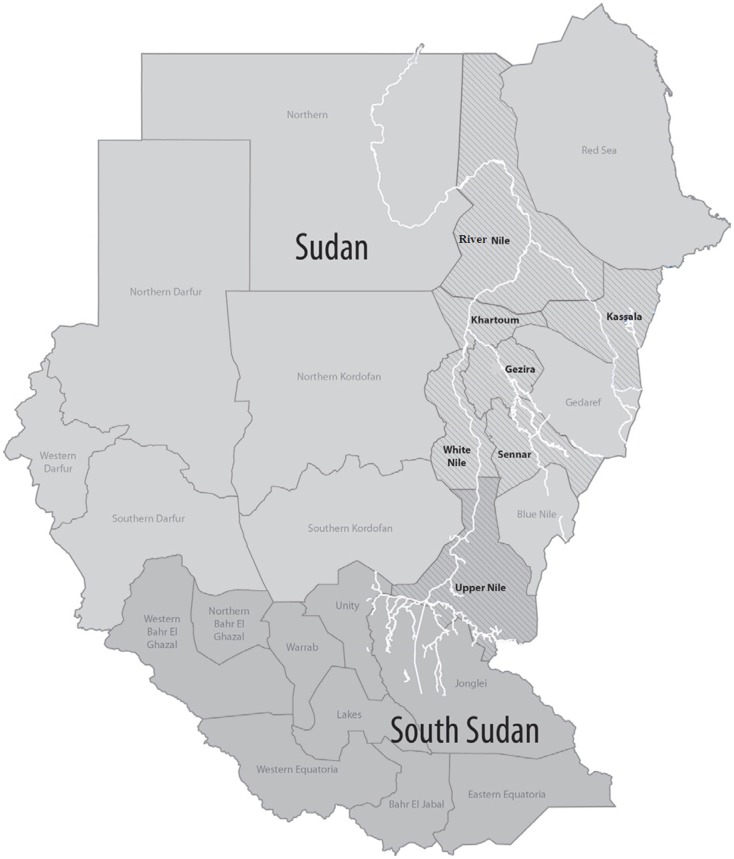
**Sudan map shows states with confirmed Rift Valley fever cases are in boldface during 2007 and 2010 outbreaks**. Source: Aradaib et al. (8), with permission from Stuart T. Nichol, Centers for Disease Control and Prevention, Atlanta, GA, USA.

The RVF has demonstrated capacity for emerging in new territories or for re-emerging after long periods of silence. Since the first outbreak in 1915, epizootics occurred periodically in Kenya until the disease was recognized in South Africa in 1951 (60), when humans became ill after handling dead and infected animals (3). Further, RVF outbreaks have been confirmed in most sub-Saharan countries (61) moving through the Rift Valley from Kenya to Tanzania, Zimbabwe, Zambia, and subsequently, RVF outbreak was first recorded in 1987 in West Africa in Senegal and Mauritania (3). RVF spread northwards through the Nile Valley into Southern Sudan and to White Nile state in Sudan where the first outbreak was identified in 1973. The disease then spread among other neighboring states within the country (Figure 2) and up to the Egyptian delta where a major epidemic with 20,000–200,000 clinical illnesses and 600 deaths was reported in 1977 (3, 62, 63). The disease also spread from continental Africa to Madagascar in 1991 (64–67) and to the Arabian Peninsula in Saudi Arabia and Yemen in 2000 (4). The recent RVF outbreaks in East Africa in Somalia (2006–2007) (5), Kenya (2006–2007) (6), Tanzania (2007) (5), and Sudan (2007–2008) (7) showed the changing epidemiology of the disease from being originally associated with livestock to infecting humans considerably and resulting in high-fatality rates (7).

**Figure 3:**
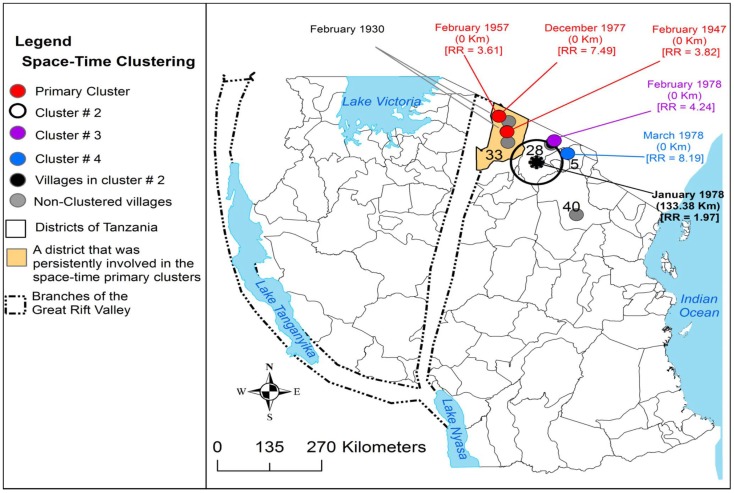
**Distribution of village-level space-time clusters of RVF cases from 1947 to 1978**. The authors set model parameters for maximum spatial and temporal window sizes and that such cluster could include a maximum of 50% of all cases. They indicated there were no clusters detected in 1930, from 1947 to 1978 three primary clusters were persistently detected in Ngorongoro district, each involving one village. An asterisk represents the center of cluster that involved more than one village; relative risk for each cluster is displayed (RR) along with the buffer (circle) size in kilometers (km). Source: Sindato et al. (68), with permission from Calvin Sindato, National Institute for Medical Research, Tabora, Tanzania.

**Figure 4:**
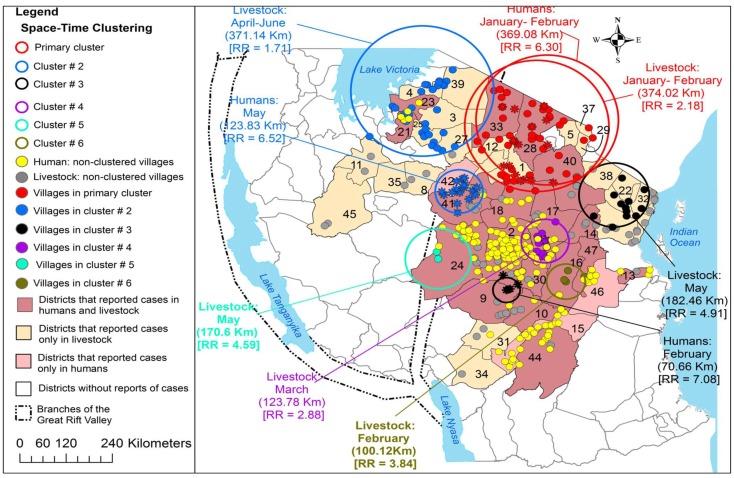
**Distribution of village-level space-time clusters of RVF cases in humans and domestic ruminants**. The authors set model parameters for maximum spatial and temporal window sizes and that such cluster could include a maximum of 50% of all cases. They conducted the analysis of clustering of cases separately for humans and domestic ruminants during the 2006/2007 outbreak wave. Between January and February 2007, there was an overlap of livestock and human primary clusters in the same location. Asterisks correspond to villages that were included within human space-time clusters; relative risk for each cluster is displayed (RR) along with the buffer (circle) size in kilometers (km). Source: Sindato et al. (68), with permission from Calvin Sindato, National Institute for Medical Research, Tabora, Tanzania.

Sindato and others investigated the spatial and temporal pattern of RVF outbreaks in Tanzania over the past 80 years (68). All RVF outbreaks reported during 1930–2007 were found to occur between December and June. Expansion of the disease into new geographical areas from the original documented outbreaks was observed. For example, between 1930 and 1957 only <1% of the districts in Tanzania were repeatedly involved in the outbreaks (Figure 3). The 1977–1978 outbreak wave had involved 3.33% districts. A relatively larger outbreak wave in 1997–1998 involved 7.70% of the districts and the widespread outbreak in 2006–2007 involved humans and domestic ruminants in 39.17% of the districts in the country (Figure 4). However, despite this expansion into districts, which were not involved before, RVF outbreaks still show significant spatio-temporal clustering in eastern Rift Valley during the last 80 years in Tanzania (68). The space-time clustering of livestock and human cases showed a tendency to spread from the north to east-central and western parts of the country (Figures 3 and 4). Uncontrolled livestock movement has been suggested as being responsible for the geographical expansion and the cumulative effect of the amount of rainfall was considered the main cause of the outbreaks (68–72). It has been suggested that the bimodal rainfall pattern experienced in this ecosystem provides an environment for *Aedes* mosquito species to emerge in large numbers at the onset of the rainy season, and therefore, resulting in extensive biting rates and transmission of the virus in animals and humans (68).

It is well established that RVFV outbreaks occur predominantly after unusual flooding events. *Aedes* mosquito species are seen as reservoir, as well as vectors, since their transovarially infected eggs withstand desiccation and larvae emerge when the eggs get into contact with water (37, 73). Transovarial transmission is assumed as the mechanism of virus persistence between epizootic events. After flooding, the infected *Aedes* mosquito eggs will hatch in the persisting water collections, and develop into infective adult mosquitoes. A study in the Ferlo region of Senegal in 2003 observed that when the rainy season began with heavy rains, the temporary ponds that serve as the breeding sites for mosquitoes were flooded to their maximum level immediately. As a result, *A. vexans arabiensis* populations were found to be abundant at the very beginning of the season, when the majority of eggs in quiescence were flooded (74). The effect of flood water on *Aedes* breeding habitats has also been studied artificially in central Kenya by sequentially flooding such habitats to determine the numbers of mosquito eggs hatching during each flooding. The authors documented that approximately 90% of the larvae sampled during four flooding events emerged during the initial one (75). This probably explains why excessive rainfall can result in high density of initial infected population. This hypothesis was supported by the study from Senegal, which found that female mosquitoes hatching from eggs laid during the previous year quickly laid eggs on the pond’s wet soil (74). The study also observed that during rainless periods lasting longer than 7 days, the time needed for embryogenesis, these new eggs undergo dormancy as the water level goes down. Once, the rains fall again, large numbers of new eggs hatch resulting into an increase in population, and thereby suggesting that several generations of infected adults can exist during the same rainy season. This dynamic has been seen to also maximize the virus’ chance to persist from one year to another in high-stock population, thus, facilitating endemisation of RVFV that is then amplified through feeding of infected adult female mosquitoes on wild and domestic ungulates and may reach epizootic and epidemic dimensions (42, 74).

**Figure 5:**
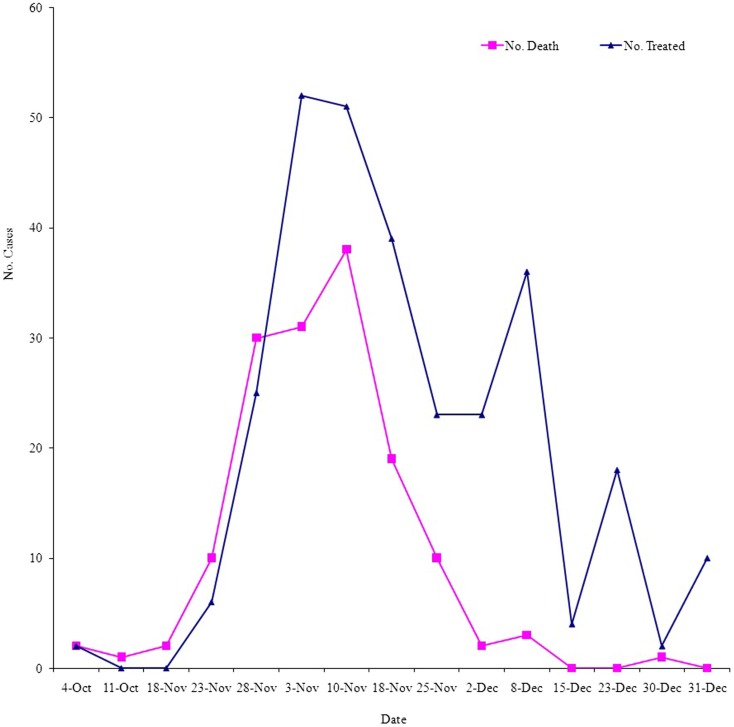
**Cases (No. received the treatment) and deaths from RVF over the period of the outbreak in Gezira State, Sudan, September–December 2007**. Source: Epidemiology Unit, Ministry of Health, Gezira State.

This dynamic can be observed from the 2007 RVF outbreak in Gezira State, Sudan (Figure 5), when satellite monitoring (June–September, 2007) showed that most of the central Sudan could be unusually subjected to heavy rainfall (76). Accordingly, a RVF risk warning has been generated for central and southern Sudan. Indeed, the predicted unusually heavy rains occurred during July–August and resulted in severe floods (77). In September, suspected human RVF cases were reported (78). The first cases appeared in southern areas of Algabalain locality in White Nile state. The first symptoms among the suspected cases were hemorrhage and fever with rapid death. All reported cases in the beginning of the outbreak were scattered and did not reach any health facilities (21). First human index case was confirmed on 8–14 October, 2007 (76). The RVF outbreak in Sudan came to public attention on October 18, 2007 when the Federal Ministry of Health (FMoH) Sudan asked the WHO to assist in the investigation and control of a suspected hemorrhagic fever. The WHO and FMoH teams started investigations in the White Nile state, central Sudan, on October 24, and on the basis of initial results, an outbreak of RVF was declared on October 28, and more help was requested for control measures (79). An announcement was made regarding RVF in animals (80) when the outbreak reached its peak in humans by early November (Figure 5). At the end of the outbreak as of late December 2007 to January 2008, a cumulative total of 698 cases, including 222 deaths, was reported from six states (Gezira, Kassala, Khartoum, River Nile, Sennar, and White Nile), yielding an overall CFR of 31.8%. This RVF outbreak was the first one reported in humans and connected directly to heavy rainfall, flooding, and increase in mosquito breeding sites in Sudan (81).

The RVF outbreak of 2007 in Sudan not only validated the association between abnormal rainfall and RVF outbreak but also prediction of RVF outbreak and early warning signs from satellite monitoring. This also showed that the wave of RVF outbreak is likely to end as the water pools due to rainfall and warm temperatures faded-out (68). This is indeed the case as there is only one short-rainy season in Sudan, which ends in October and then the winter season begins at the end of November and runs up to late February. The presumed link between extraordinary flooding events and RVF outbreaks was also well validated, among others, by a successful prediction of the 2007 outbreak in Somalia, Kenya, and northern Tanzania, using climate modeling (82). In fact, each of the seven documented moderate or large RVF outbreaks that have occurred in East Africa over the last 60 years have been associated with El Niño Southern Oscillation (ENSO) associated with above normal and widespread rainfall (83). This association of RVF with excessive rainfall and flooding was also observed in other countries outside the African continent in Arabian Peninsula, i.e., the outbreak of 2000 in Yemen (84).

Interestingly, all RVF outbreaks in Sudan originated in White Nile State where the first RVFV was identified in 1973 as the cause of an extensive epizootic (10), then moved northward through the White Nile river valley to Khartoum in 1976 (85) and extended to the neighboring states of Gezira, Sennar, and Kassla states during 2007 outbreak (Figure 1). In the White Nile State, the river Nile has a very wide basin, which floods annually between June and September, resulting in wide wetland along the valley of the river from the border of Southern Sudan up to Khartoum. It is important to mention that the five states are located in the Central Clay Plain soil of the Sudan, which extends from west of Kassala through Gezira, Khartoum, White Nile up to southern Kurdufan. This type of soil and topography when flooded create large shallow wetlands similar to what is known as “*dambo*,” which is often shown as suitable breeding habitats for *Aedes* mosquitoes in central, southern, and eastern Africa. This suggested that local environment is very important and is directly linked to RVF outbreak. Significant association was observed between RVF outbreaks from 1930 to 2007 and clay and loam soil textures in the eastern Rift Valley ecosystem of Tanzania where clustering of RVF outbreaks were persistently and predominantly detected (68). Clay soil rather than sandy soil texture supports long-period retention of water contributing to flooding and wetness of habitat suitable for breeding and survival of *Aedes* mosquito vectors. This suggests that while rainfall might be the major determinant for the onset and switch-off of an outbreak, it is unlikely that it is the only factor responsible for the spread and clustering of RVF cases. A causal association between local environmental factors, livestock density and movement, encroachment of mosquitoes into new areas, and occurrence of RVF has been suggested in previous studies (20, 86, 87).

## Risk Factors during RVF Outbreak

It is generally accepted that during the 2007 outbreak in Sudan, animal contact was the most dominant risk factor followed by animal products and mosquito bites (78). This is supported by the fact that the 2010 outbreak was first characterized by abortions in ewes followed by infections in persons with histories of contact with aborted fetal material (8). Contact with RVFV-infected animals such as consuming or handling products from sick animals, touching an aborted animal fetus, or being a herdsperson has been documented as the most important risk factor for severe infection during the 2007 outbreak in Kenya (76). A similar result was observed during the previous RVF outbreak of 1997–1998 in northern Kenya (11). These findings are consistent with those from another study from Sudan stating that most of the animals such as sheep, cattle, goats, and camels stay very close to their owners’ houses at night (21, 88).

Despite the fact that there was no evidence for horizontal transmission between humans in Sudan or elsewhere, risk from infected pregnant women through vertical transmission can occur. During the 2007 outbreak in Sudan, a 29-year-old pregnant woman presented in early labor with symptoms suggestive of RVF and delivered a baby weighing 3.2 kg with skin rash, palpable liver, and spleen. Two samples from the mother and neonate were screened and found to be positive for RVF-IgM (89). This case demonstrated that RVF can be vertically transmitted in human. A similar case was also reported before in Saudi Arabia, during the 2000 outbreak (90). These are consistent with the claims recently made about the burden of emerging zoonotic infectious disease among women in general and pregnant women, in particular (91).

Movement of animals during an outbreak can be a serious risk factor. Complete genome sequences from RVFV strains detected during the 2007 and 2010 outbreaks in Sudan suggested multiple introductions of RVFV into Sudan as part of sweeping epizootics from eastern Africa (8). All RVFV strains observed grouped into Kenya-1 or Kenya-2 sub lineages, which defined the eastern Africa outbreak in 2006–2008 (92). The sequencing also suggested that an earlier common ancestor from 1996 coinciding with the 1997–1998 outbreaks in the horn of Africa. The Kenya-2 sub lineage is now known to be widely distributed in Tanzania and Sudan (8, 92, 93). The movement of animals from southern states in White Nile to northern ones in Gezira, Khartoum, and Kassala for marketing was most likely responsible for the geographical expansion of the virus in central and eastern Sudan (Figure 2). Identical or nearly identical sequences of the virus strains were identified for different states and years, Khartoum in 2007 and Gezira in 2010, as well as Khartoum and West Nile in 2007. These sequences indicate recent movement of the virus in this region and support the necessity and utility of surveillance systems for recognizing when and where a large epidemic is imminent (8).

## Surveillance and Control of RVF Outbreak: The Example of Saudi Arabia in 2000

Saudi Arabia and Yemen experienced a huge RVF outbreak in the year 2000 (29, 30, 54, 55). It was the first outbreak in Middle East outside its endemic areas in Africa. The outbreak in Saudi Arabia is suggested to have been from eastern Africa by importation of infected animals (40), similar to the suggested route of introduction of RVFV into Egypt in 1977 from Sudan (94). The virus causing the Saudi Arabia outbreak belonged to the same strain that caused the 1997–1998 outbreaks in East Africa (95).

After the outbreak was declared, a team was established in collaboration between the Ministries of Health, Agriculture, and Water, and the Ministry of Municipalities and international organizations such as CDC, WHO, and National Institute of Virology, South Africa, to control the outbreak (30, 54, 96). A strategy called “One Health” was then implemented by Saudi Arabia targeting both the animal and human hosts (17). The urgent integrated control measures that were implemented by this strategy during the outbreak included the following activities: (1) disposal of dead animals in an appropriate manner, (2) active surveillance surveys to detect cases of RVF among humans and animals to locate target areas for animal vaccination, and (3) apply a vaccination campaign that started in October 2000 (16, 30, 31, 54, 96). Around 1,200,000 doses of the vaccine were reported to be imported into Saudi Arabia and the campaign continued in 2001 with more than 10 million ruminants being vaccinated (31). These activities were accompanied by (4) a restriction on animal movements outside the affected areas and a ban on animal imports from RVF-enzootic countries (29). (5) For effective case management, detailed case definition was developed, training sessions on how to manage the suspected cases clinically was implemented, two well-prepared laboratories (one in the affected regions and the other in the capital of the country) for diagnosis of RVFV antibodies in suspected cases were also provided by the Saudi Ministry of Health (29, 54, 96). (6) Epidemiological investigation was also performed to identify risk factors (30). (7) In addition, an entomological study to search for the mosquito breeding grounds (30) was followed by an intensive mosquito control program with spraying (54, 97). This strategy succeeded to limit the effect of the outbreak and curb the disease from spreading to other areas. Since 2000, only sporadic cases have been recorded in Saudi Arabia and only in the same regions where the original outbreak was reported (16). Later investigation on this strategy concluded that “One Health” approach is the best option to mitigate outbreaks of RVF. Collaboration between veterinary, health, and environmental authorities both at national and regional levels is needed to control RVF outbreak (17).

## Conclusion and Perspective

From the foregoing narrative, we can conclude that RVF causes huge health and economic losses signified by the number of human deaths and high mortality and abortion rates in livestock. It is also clear that whereas RVF was previously restricted to specific areas in sub-Saharan Africa, the disease seems to be spreading into new territories beyond the traditional foci as evidenced by outbreaks in the Arabian Peninsula. The epidemiology of RVF is complex and transmission involves multiple mosquito vector species. A multiplicity of factors shapes the epidemiology of RVF. Key among these is rainfall and flooding, soil types, contact with animals, breeding sites, and availability and movement of livestock. Epizootics are interspaced with long periods of quiescence.

It is our considered view that repeated outbreaks could be forestalled with adequate sensitization of the policy makers. It is also clear that with enhanced coordination among stakeholders, e.g., Ministries of Health and Livestock, researchers, and local communities it is possible to better handle future outbreaks. Such coordination of stakeholders seems to have worked effectively in managing the outbreak in Saudi Arabia. Other regions such as eastern Africa that has borne the brunt of previous outbreaks should learn from the Saudi experience. In light of improved warning signs derived from satellite imagery and mapping, governments should come up with clear strategies and action plans for preparedness and handling of future outbreaks. Such strategies should include strong surveillance systems, adequate and well trained personnel, among others.

## Author Contributions

Yousif E. Himeidan suggested the topic, framed, drafted, and wrote up the manuscript. Mostafa M. Mahgoub collected the data on patients from Ministry of Health, Gezira State. Eliningaya J. Kweka, El Amin El Rayah, and Johnson O. Ouma drafted and reviewed the manuscript. All authors read and approved the final version.

## Conflict of Interest Statement

The authors declare that the research was conducted in the absence of any commercial or financial relationships that could be construed as a potential conflict of interest.
